# ECHO Elimination of Cervical Cancer in Latin America (ECHO ELA): Lessons Learned From Promoting WHO’s Cervical Cancer Elimination Goals ‘90-70-90’

**DOI:** 10.1200/GO.22.12000

**Published:** 2022-05-05

**Authors:** Melissa Lopez Varon, Mila Pontremoli Salcedo, Kathleen Schmeler, Sandra San Miguel-Majors, Edward L. Trimble, Doug Lowy, Silvina Arrossi, Maria Tereza da Costa Oliveira, Mauricio Maza, Jane Montealegre, Lucia Helena de Oliveira, Sara Benitez-Majano, Silvana Luciani

**Affiliations:** ^1^ The University of Texas MD Anderson Cancer Center, Houston, TX; ^2^ National Cancer Institute, Rockville, MD; ^3^ Instituto Nacional de Cancer de Argentina, Buenos Aires, Argentina; ^4^ PAHO/WHO, Washington, DC; ^5^ Basic Health International, San Salvador, El Salvador and New York, NY; ^6^ Baylor College of Medicine, Houston, TX

## Abstract

Cervical cancer (CC) is one of the leading causes of cancer deaths among women in some countries in the Region of the Americas.^1^ CC is preventable through HPV vaccination, screening and treatment of precancerous lesions, and can be effectively treated if diagnosed early.

**METHODS:**

Under the framework of the Global Strategy to Accelerate the Elimination of CC as a Public Health Problem, The University of Texas MD Anderson Cancer Center (MD Anderson), the US National Cancer Institute (NCI), and the Pan American Health Organization (PAHO) partnered to develop the Project ECHO for the elimination of CC in Latin America (ECHO ELA). ECHO ELA is modeled on Project ECHO® (Extension of Community Healthcare Outcomes) a hub-and-spoke knowledge-sharing approach where expert teams lead virtual didactic lectures and case discussions, building capacity for participants to deliver best-practice programs to their regions.^3^ ECHO ELA consists of monthly, Spanish telementoring conferences to assist Latin American (LA) countries reaching the WHO CC elimination goals “90-70-90:” vaccinating 90% of girls against HPV by the age of 15, screening 70% of women for cervical cancer at ages 35 and 45 and treating 90% of women diagnosed with pre-invasive disease or cervical cancer. Program targets professionals from Ministries of Health, including Immunization and Screening Program Managers, and cervical cancer stakeholders.

**RESULTS:**

In the first year of the program (2020-2021), 294 participants from 22 countries were registered. Fourteen sessions were held averaging 77 participants per session on topics spanning comprehensive program planning, presenting country experiences on HPV vaccination strategies, new technologies for screening and treatment. Eighteen participants completed the post survey addressing priorities, capacity, and desired outcomes, and two focus groups collected facilitators/barriers to achieving goals.

**CONCLUSION:**

ECHO ELA is potentially an effective tool to enhance collaboration and support countries’ progress towards the elimination of CC.

